# The Prognostic Value of Haplotypes in the Vascular Endothelial Growth Factor A Gene in Colorectal Cancer

**DOI:** 10.3390/cancers2031405

**Published:** 2010-06-28

**Authors:** Torben F. Hansen, Karen-Lise G. Spindler, Rikke F. Andersen, Jan Lindebjerg, Steen Kølvraa, Ivan Brandslund, Anders Jakobsen

**Affiliations:** 1 Department of Oncology, Vejle Hospital, Vejle, Denmark; E-Mails: karen-lise.garm.spindler@slb.regionsyddanmark.dk (K.-L.G.S.); anders.jakobsen@slb.regionsyddanmark.dk (A.J.); 2 Department of Biochemistry, Vejle Hospital, Vejle, Denmark; E-Mails: rikke.fredslund.andersen@slb.regionsyddanmark.dk (R.F.A.); ivan.brandslund@slb.regionsyddanmark.dk (I.B.); 3 Department of Clinical Pathology, Vejle Hospital, Vejle, Denmark; E-Mail: jan.lindebjerg@slb.regionsyddanmark.dk (J.L.); 4 Department of Clinical Genetics, Vejle Hospital, Vejle, Denmark; E-Mail: steen.kolvraa@slb.regionsyddanmark.dk (S.K.)

**Keywords:** colorectal neoplasm, single nucleotide polymorphisms, haplotypes, vascular endothelial growth factor A, survival

## Abstract

New prognostic markers in patients with colorectal cancer (CRC) are a prerequisite for individualized treatment. Prognostic importance of single nucleotide polymorphisms (SNPs) in the vascular endothelial growth factor A (*VEGF-A*) gene has been proposed. The objective of the present study was to investigate the prognostic importance of haplotypes in the *VEGF-A* gene in patients with CRC. The study included 486 patients surgically resected for stage II and III CRC, divided into two independent cohorts. Three SNPs in the *VEGF-A* gene were analyzed by polymerase chain reaction. Haplotypes were estimated using the PHASE program. The prognostic influence was evaluated using Kaplan-Meir plots and log rank tests. Cox regression method was used to analyze the independent prognostic importance of different markers. All three SNPs were significantly related to survival. A haplotype combination, responsible for this effect, was present in approximately 30% of the patients and demonstrated a significant relationship with poor survival, and it remained an independent prognostic marker after multivariate analysis, hazard ratio 2.46 (95% confidence interval 1.49–4.06), p < 0.001. Validation was provided by consistent findings in a second and independent cohort. Haplotype combinations call for further investigation.

## 1. Introduction

Reliable prognostic markers are of great clinical importance in colorectal cancer (CRC). Several prognostic markers are already being used by clinicians to select patients for further postoperative treatment and adjuvant chemotherapy is standard of care for patients with high risk stage II and stage III CRC [1,2,3,4,5]. Nevertheless, tumor recurrence still occurs in patients with low risk stage II tumors and far from all patients receiving adjuvant chemotherapy benefit from their treatment. The identification of new prognostic markers in CRC is therefore a prerequisite for selection for adjuvant treatment in this patient category.

Angiogenesis, the development of new blood vessels from the pre-existing vasculature, is a normal physiologic phenomenon but it also represents one of the classical hallmarks in the malignant transformation of tumors [6]. The vascular endothelial growth factor (VEGF) system is one of the most essential growth factor systems involved in neoangiogenesis in malignant tumors [7]. It influences the vasculature and affects the growth of tumors [8], and it provides a potential route for the dissemination of tumor cells and thereby increases the risk of metastatic spread [9]. The biologic availability of VEGF-A, the most important ligand in the VEGF system, therefore has the potential to influence the prognosis of patients with malignant diseases.

The level of VEGF-A can, among other factors, be affected by normal sequence variations in the *VEGF-A* gene. Several of such normal variations, single nucleotide polymorphisms (SNPs), in the *VEGF-A* gene have been described. The −2578 C/A SNP (rs699947) and the −460 C/T SNP (rs833061) in the promoter region and the 405 G/C (rs2010963) in the 5’ untranslated region (5’UTR) are all very common and they are often analyzed in clinical studies. Associations between SNPs in the *VEGF-A* gene and the promoter activity of the gene and protein concentrations of VEGF-A have been demonstrated in some studies [10,11] and clinical studies have also suggested association with clinical outcome in breast [12], renal cell [13], gastric [14], colorectal [15,16,17], and ovarian cancer [18]. A high degree of linkage disequilibrium exists between several of the SNPs in the promoter and 5’UTR of the *VEGF-A* gene. It is therefore possible that a haplotype effect, rather than an individual effect of SNPs, might explain some of the prognostic information related to the genetic variations in this region of the gene. In agreement with this, haplotype analyses have supplied further information on these genetic variations [12,14,15]. Focus, however, has so far been on individual haplotypes rather than haplotype combinations. The latter may be relevant to providing specific information on the prognosis for each individual patient [19].

The literature on the prognostic importance of SNPs in the *VEGF-A* gene in patients with CRC is still rather sparse, with conflicting results [15,17], and validation studies are warranted. Furthermore, only very few studies have reported on the effect of haplotypes. The aim of the present study was to perform a comprehensive analysis of the possible prognostic importance of haplotypes in the *VEGF-A* gene in two independent cohorts of patients with stage II and III CRC. 

## 2. Results and Discussion

### 2.1. Patient Characteristics

Patient characteristics are presented in Table 1. The two cohorts were comparable. No significant differences were found comparing allele frequencies between the two cohorts, and all three SNPs were found to be in Hardy-Weinberg equilibrium (p > 0.05).

**Table 1:** Patient characteristics. Test cohort (n = 191) and validation cohort (n = 295).

	Test cohort	Validation cohort
	Number (%)	Number (%)
Sex		
Male	98 (51)	159 (54)
Female	93 (49)	136 (46)
Age (years)		
Mean (SD)	70.1 (11.6)	70.5 (11.2)
Range	31–97	33–92
pT category		
1–3	159 (83)	248 (84)
4	32 (17)	47 (16)
pN category		
0	99 (52)	163 (55)
1–2	92 (48)	132 (45)
Stage		
II	98 (51)	163 (55)
III	93 (49)	132 (45)
Localization		
Colon	121 (63)	197 (67)
Rectum	70 (37)	98 (33)
Vascular invasion		
Yes	11 (9)	35 (12)
No	111 (91)	249 (88)
Neuronal invasion		
Yes	11 (9)	31 (12)
No	109 (91)	236 (88)
Peritoneal perforation		
Yes	26 (14)	38 (13)
No	160 (86)	253 (87)
Adjuvant chemotherapy		
No	157 (82)	232 (81)
Yes	34 (18)	53 (19)
5-FU/Leucovorin or capecitabine	34 (100)	28 (53)
XELOX	0 (0)	25 (47)
−2578 C/A SNP		
CC	47 (25)	80 (27)
CA	85 (45)	147 (50)
AA	59 (31)	68 (23)
−460 C/T SNP		
CC	59 (31)	69 (23)
CT	85 (45)	146 (49)
TT	47 (25)	80 (27)
405 G/C SNP		
GG	95 (50)	128 (43)
GC	74 (39)	129 (44)
CC	22 (12)	38 (13)
Sum of the percentages do not always equal 100 % due to rounding of data. Not all patients had complete patient characteristics available.		

The median disease free survival (DFS) was 6.4 years (95% CI, 4.6–8.8) and the median overall survival (OS) was 7.2 years (95% CI, 5.3–9.3) in the test cohort. The median DFS was 5.8 years (95% CI, 5.1–5.8) in the validation cohort. The median OS was not reached in the validation cohort but does exceed 7.0 years. One hundred and two patients from the test cohort and 67 patients from the validation cohort died. Follow-up ended December 15, 2009 and data are reported with a median observation time of 9.8 years (range, 3.1–10.9) in the test cohort and 3.4 years (range, 1.0–7.8) in the validation cohort.

No significant associations were found between the genotypes of the three SNPs and patient characteristics, as listed in Table 1 in either of the cohorts Tumors with the T categories 1, 2 and 3 were grouped together due to the presence of very few T_1_ and T_2_ tumors. 

The standard prognostic markers, T and N category, stage, vascular invasion and peritoneal perforation were all significantly associated with survival in both cohorts (data not shown).

### 2.2. The Prognostic Value of VEGF-A SNPs

Table 2 shows the relationship between SNP status and survival. The striking finding here is that the heterozygous VEGF-A genotypes (−2578 CA, −460 CT, and 405 GC) were all related to inferior survival rates compared to the corresponding homozygous genotypes in the test cohort. A comparison of the heterozygous genotypes to both homozygous genotypes was consequently performed although such a comparison differs from the conventional way of dividing genotypes. This strategy was kept in the following analyses in the validation cohort. The −460 C/T SNP were significantly related to DFS and OS in both cohorts. 

**Table 2:** Relationship between SNP status and survival in both cohorts. All patients are included.

**Disease free survival (DFS)**									
**Test cohort**						**Validation cohort**			
VEGF-A SNP and genotype comparison*	**Events**	**HR (95% CI)**	**p-value**		VEGF-A SNP and genotype comparison*		**Events**	**HR (95% CI)**	**p-value**
−2578 C/A					−2578 C/A				
CC (NR) v CA (3.3)	19 v 55	0.47 (0.30–0.75)	<0.01		CC (5.6) v CA (5.8)		17 v 49	0.62 (0.37–1.03)	0.09
CC (NR) v AA (8.5)	19 v 29	0.83 (0.47–1.46)	0.52		CC (5.6) v AA (NR)		17 v 15	1.13 (0.57–2.26)	0.73
CA (3.3) v AA (8.5)	55 v 29	1.78 (1.16–2.74)	0.01		CA (5.8) v AA (NR)		49 v 15	1.75 (1.05–2.93)	>0.05
CA v CC + AA		1.93 (1.29–2.89)	<0.01		CA v CC + AA			1.68 (1.09–2.60)	0.02
−460 C/T					−460 C/T				
CC (8.5) v CT (3.3)	29 v 55	0.56 (0.37–0.86)	0.01		CC (NR) v CT (5.8)		15 v 50	0.55 (0.33–0.91)	0.04
CC (8.5) v TT (NR)	29 v 19	1.21 (0.68–2.14)	0.52		CC (NR) v TT (5.6)		15 v 16	0.91 (0.45–1.85)	0.80
CT (3.3) v TT (NR)	55 v 19	2.11 (1.33–3.35)	<0.01		CT (5.8) v TT (5.6)		50 v 16	1.73 (1.05–2.87)	>0.05
CT v CC + TT		1.93 (1.29–2.89)	<0.01		CT v CC + TT			1.78 (1.15–2.75)	0.01
405 G/C					405 G/C				
GG (7.5) v GC (4.3)	50 v 43	0.73 (0.48–1.11)	0.13		GG (NR) v GC (5.0)		24 v 48	0.41 (0.26–0.65)	<0.01
GG (7.5) v CC (NR)	50 v 10	1.16 (0.61–2.22)	0.67		GG (NR) v CC (NR)		24 v 9	0.69 (0.30–1.60)	0.34
GC (4.3) v CC (NR)	43 v 10	1.55 (0.84–2.85)	0.21		GC (5.0) v CC (NR)		48 v 9	1.66 (0.90–3.06)	0.16
GC v GG + CC		1.40 (0.93–2.12)	0.09		GC v GG + CC			2.22 (1.42–3.46)	<0.01
**Overall survival (OS)**									
**Test cohort**						**Validation cohort**			
VEGF-A SNP and genotype comparison*	**Events**	**HR (95% CI)**	**p-value**		VEGF-A SNP and genotype comparison*		**Events**	**HR (95% CI)**	**p-value**
−2578 C/A					−2578 C/A				
CC (NR) v CA (4.7)	18 v 55	0.46 (0.29–0.74)	<0.01		CC (6.1) v CA (NR)		12 v 40	0.55 (0.31–0.97)	0.06
CC (NR) v AA (8.8)	18 v 29	0.79 (0.44–1.40)	0.42		CC (6.1) v AA (7.0)		12 v 15	0.84 (0.39–1.79)	0.65
CA (4.7) v AA (8.8)	55 v 29	1.71 (1.12–2.63)	0.02		CA (NR) v AA (7.0)		40 v 15	1.38 (0.79–2.41)	0.28
CA v CC + AA		1.89 (1.27–2.82)	<0.01		CA v CC v AA			1.57 (0.97–2.54)	0.07
−460 C/T					−460 C/T				
CC (8.8) v CT (4.7)	29 v 55	0.58 (0.38–0.90)	0.02		CC (7.0) v CT (NR)		15 v 41	0.69 (0.40–1.20)	0.22
CC (8.8) v TT (NR)	29 v 18	1.27 (0.72–2.26)	0.42		CC (7.0) v TT (NR)		15 v 11	1.26 (0.58–2.72)	0.56
CT (4.7) v TT (NR)	55 v 18	2.15 (1.35–3.43)	<0.01		CT (NR) v TT (NR)		41 v 11	1.99 (1.13–3.53)	0.04
CT v CC + TT		1.89 (1.27–2.82)	<0.01		CT v CC + TT			1.66 (1.03–2.68)	0.04
405 G/C					405 G/C				
GG (8.3) v GC (4.8)	50 v 43	0.72 (0.47–1.10)	0.11		GG (NR) v GC (5.8)		23 v 39	0.51 (0.31–0.84)	0.01
GG (8.3) v CC (NR)	50 v 9	1.33 (0.70–2.54)	0.43		GG (NR) v CC (NR)		23 v 5	1.21 (0.49–3.02)	0.70
GC (4.8) v CC (NR)	43 v 9	1.82 (0.99–3.35)	0.10		GC (5.8) v CC (NR)		39 v 5	2.25 (1.11–4.58)	0.08
GC v GG + CC		1.46 (0.97–2.20)	0.06		GC v GG + CC			2.00 (1.23–3.25)	<0.01

* Median DFS and OS in years are given in parentheses. CI: confidence interval; NR: not reached.

The poor survival related to the heterozygous genotypes in the univariate analyses was also seen in the multivariate analyses. This is presented in Table 3, which shows the results of the multivariate survival analysis from the validation cohort. The possible independent prognostic value of each SNP was assessed here individually. The table therefore summarizes the results from three separate Cox regression models all including T category, N category, vascular invasion, neuronal invasion, peritoneal perforation and adjuvant treatment. The −2578 C/A and the 405 G/C SNPs both demonstrated prognostic value independent of the standard prognostic markers regarding DFS. A possible independent value, although not significant, was seen for the −460 C/T SNP. A similar multivariate survival analysis adjusted for the same variables was initially performed on the test cohort, with similar findings although the 405 G/C SNP failed to demonstrate an independent prognostic value (data not shown).

**Table 3:** Multivariate survival analysis according to the VEGF-A SNPs. Validation cohort, n = 295.

Genotypes	Disease free survival (DFS)		Overall survival (OS)	
	HR (95% CI)	p-value	HR (95% CI)	p-value
−2578 C/A				
CC	1		1	
CA	1.98 (1.10–3.56)	0.02	1.92 (0.98–3.76)	0.06
AA	1.10 (0.53–2.28)	0.80	1.32 (0.60–2.92)	0.49
−460 C/T				
CC	1		1	
CT	1.81 (0.99–3.31)	0.06	1.46 (0.79–2.72)	0.23
TT	0.89 (0.42–1.87)	0.76	0.73 (0.32–1.64)	0.44
405 G/C				
GG	1		1	
GC	1.79 (1.06–3.01)	0.03	1.52 (0.88–2.63)	0.13
CC	0.98 (0.43–2.26)	0.97	0.73 (0.27–1.98)	0.53

The VEGF-A SNPs were added individually to the multivariate analysis and the hazard ratios therefore represent the results from three separate Cox regression models all including T category, N category, vascular invasion, neuronal invasion, peritoneal perforation and adjuvant treatment. HR: hazard ratio; CI: confidence interval.

### 2.3. Haplotype Analysis

The haplotype analysis was performed to evaluate a possible combined effect of the SNPs (−2578 C/A, −460 C/T, 405 G/C) on CRC survival. Three haplotypes with a frequency above 0.5%, (ACG, 52.3%; CTC, 31.3%; CTG, 16.4%) in the test cohort and (ACG, 47.8%; CTC, 34.7%; CTG, 17.0%) in the validation cohort, were defined by the PHASE program based on population frequencies of the three SNPs. This meant that each patient could be identified by one of six possible haplotype combinations due to our bi-allelic nature. 

**Figure 1:**
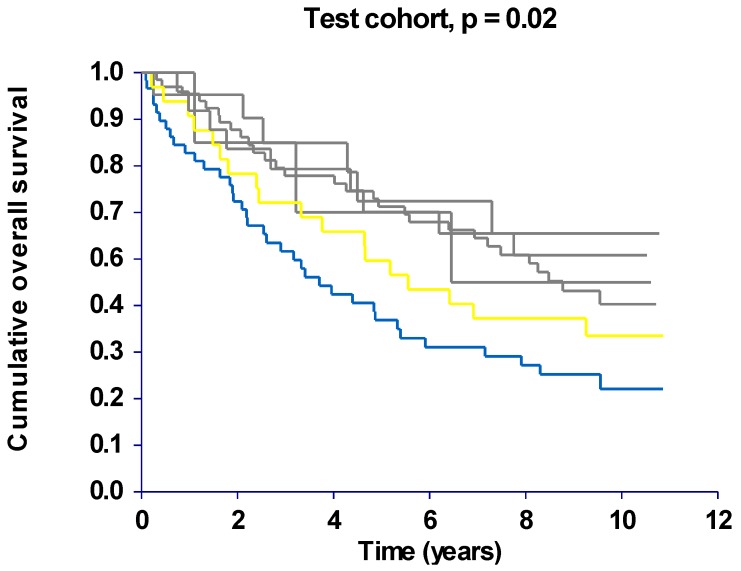
Kaplan-Meier overall survival curves according to haplotype combinations. The blue line represents patients with the (CTC, ACG) haplotype combination and the yellow line patients with the (CTG, ACG) combination. Patients with the remaining four haplotype combinations are represented by the grey lines, test cohort, n = 191.

The initial analyses in the test cohort revealed a combination (CTC, ACG) present in 29% of the patients, related to inferior survival rates and differing significantly from four of the remaining five combinations. The survival rates for these four combinations, constituting 55% of the patients, were very similar and did not differ significantly from each other. The patients with the last combination (CTG, ACG) presented with an intermediate prognosis not differing significantly from any of the other combinations. This is illustrated graphically in Figure 1 showing the OS curves from the test cohort according to the haplotype combinations. Similar DFS curves were seen (data not shown) and it therefore seemed reasonable to pool the patients with haplotype combinations different from the (CTC, ACG) and (CTG, ACG) combinations in one group for the following analyses in the validation cohort. 

**Figure 2:**
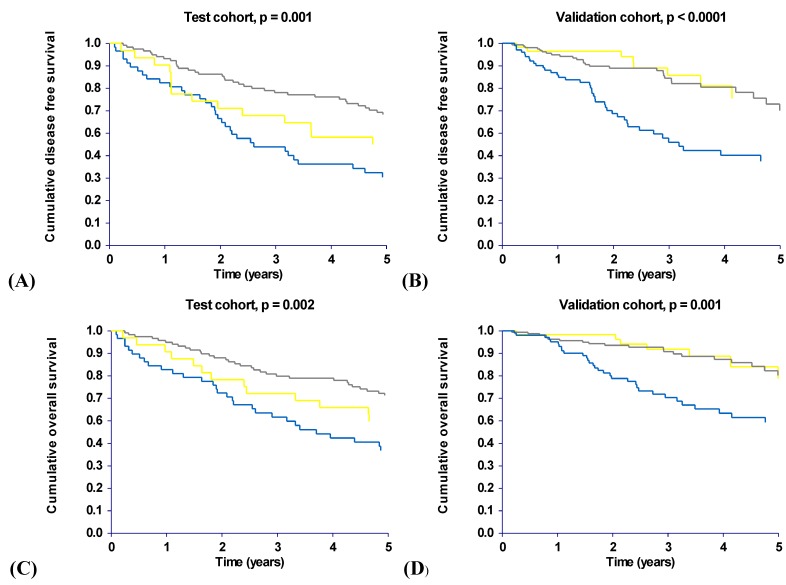
**A** to **D** Kaplan-Meier survival curves according to haplotype combinations. The blue line represents patients with the (CTC, ACG) haplotype combination and the yellow line patients with the (CTG, ACG) combination. Patients with the remaining four haplotype combinations are represented by a single grey line. All patients from both cohorts are included.

Figure 2 shows the significant differences in DFS and OS seen in both cohorts based on the above considerations. The results from the validation cohort confirmed the inferior survival rates seen in the test cohort for the patients with the (CTC, ACG) combination and furthermore the favorable survival rates seen for the combined group. The median DFS for the patients with the (CTC, ACG) combination was 2.6 years (95% CI, 2.0–4.4) in the test cohort compared to 3.2 years (95% CI, 2.2–5.8) in the validation cohort. The patients from the combined group presented with a median DFS of 9.5 years (95% CI, 6.5–9.5) in the test cohort. The median DFS for this group was not reached in the validation cohort but exceeds 5.5 years. The results from the validation cohort could not confirm the intermediate prognosis for the patients with the (CTG, ACG) combination who presented with survival rates similar to the survival rates seen for the combined group and consequently, only the (CTC, ACG) combination seems to be related to poor survival.

Table 4 shows the results of the multivariate survival analysis according to the haplotype combinations, and involves the same patient characteristics as in Table 3. The (CTC, ACG) haplotype combination remained an independent prognostic marker in both cohorts with hazard ratios ranging from 2.04 to 2.46, respectively. 

We also performed subgroup analyses running separate survival analyses for patients with colon and rectal cancers and for patients with stage II and III disease. These results did not indicate that the present findings were more pronounced in either of the groups (data not shown).

**Table 4:** Multivariate survival analysis according to haplotype combinations in both cohorts. All patients are included.

**Disease free survival (DFS)**						
**Test cohort**				**Validation cohort**		
	**HR (95% CI)**	**p-value**			**HR (95% CI)**	**p-value**
Remaining combinations	1			Remaining combinations	1	
(CTG, ACG)	1.85 (0.95–3.62)	0.07		(CTG, ACG)	0.98 (0.44–2.18)	0.97
(CTC, ACG)	2.04 (1.14–3.67)	0.02		(CTC, ACG)	2.46 (1.49–4.06)	<0.01
**Overall survival (OS)**						
**Test cohort**				**Validation cohort**		
	**HR (95% CI)**	**p-value**			**HR (95% CI)**	**p-value**
Remaining combinations	1			Remaining combinations	1	
(CTG, ACG)	1.72 (0.89–3.36)	0.11		(CTG, ACG)	0.95 (0.40–2.21)	0.90
(CTC, ACG)	2.15 (1.21–3.82)	0.01		(CTC, ACG)	2.07 (1.20–3.57)	0.01

The Cox regression models all included T category, N category, vascular invasion, neuronal invasion, peritoneal perforation and adjuvant treatment, HR; hazard ratio, CI; confidence interval.

### 2.4. Discussion

New prognostic markers are a prerequisite for selecting patients with CRC for adjuvant treatment. A number of recent publications have suggested that SNPs in the *VEGF-A* gene are biomarkers of prognostic importance in different types of malignant tumors including CRC [15,16,17]. However, the results of these studies are contradictory, probably because of varying patient materials, and the role of haplotypes needs to be elucidated. Therefore, the aim of this study was to analyze the prognostic influence of haplotypes in the *VEGF-A* gene in patients with stage II or III CRC based on a test and a validation cohort.

The three SNPs in the present study were chosen, as they are very common and because previous studies have reported on functional as well as prognostic influences [10,11,12,13,14,15,16,17,18]. Furthermore, they are all located in rather close proximity in a region of the gene in which a high degree of linkage disequilibrium exists and haplotype effects might therefore be a possibility. The genotype frequencies of these SNPs in our study are in rather good agreement with those reported in the literature on CRC patients [15,20,21,22]. 

The −2578 C/A, the −460 C/T and the 405 G/C SNPs all showed a significant relationship with survival, and in all three cases the heterozygous genotypes were related to poor survival. We know that comparing heterozygous *versus* both homozygous is not the conventional way of grouping genotypes but the present results called for this unconventional approach. Multivariate analysis of DFS (performed with each SNP individually) confirmed a significant prognostic value related to the −2578 C/A and 405 G/C SNPs independent of standard prognostic markers. Kim *et al.* examined the prognostic influence of VEGF-A SNPs in 445 Korean patients operated for CRC stage I to IV [15]. The results suggested that compared to the other genotypes the VEGF-A 405 GG genotype was associated with inferior survival rates. The −2578 C/A SNP did not show any relationship with survival. A second study, by Dassoulas *et al*. reported that the VEGF-A −2578 AA and 405 CC genotypes were related to significantly lower OS in 312 Greek patients operated for CRC stage I to IV. The influence by the −460 C/T SNP did not reach statistical significance [17]. The results from our study, the Greek, and the Korean studies, clearly demonstrate the diversity between studies dealing with the prognostic importance of SNPs. First of all, any difference in the allelic frequencies and the phenotypic outcome, survival, would make it difficult to compare the prognostic value of genetic markers between ethnically different groups. Furthermore, differences in the disease stages included in the studies might also explain some of the discrepancies. Stage is a very strong prognostic parameter, and the fraction of patients with stage I and IV disease differed a great deal between the Greek and Korean studies. We only included patients with stage II and III disease because this is the group of patients in which new prognostic markers are most warranted. 

As expected, we observed rather similar results for all three SNPs presumably due to the high degree of linkage disequilibrium known to exist in this region of the gene. The link between the −2578 C/A and the −460 C/T SNPs actually turned out to be stronger than initially expected. The distribution of genotypes was identical in the test cohort and only differed in a small number of patients in the validation cohort. Therefore, it can not be ruled out that an underlying haplotype effect can explain some of the apparent prognostic power associated with the genetic variations in this region of the gene. 

The haplotype frequencies from the present study were in a rather good agreement with the haplotype frequencies reported in the literature [15,17,22,23]. Any differences could very well be explained by ethnical differences, but also the source of DNA could account for some of the differences. The Korean studies are based on tumor DNA, where the presence of loss of heterozygosity (LOH) would lead to changes in the observed frequencies of the haplotypes.

The present haplotype analysis identified three frequent haplotypes and all patients could consequently be identified by one of six haplotype combinations. The haplotype analysis demonstrated a significant relationship between poor survival rates and patients with the (CTC, ACG) combination compared to survival rates for the remaining patients. A group of patients (CTG, ACG) with a presumable intermediate prognosis was identified in the test cohort, but this was not confirmed by the following analysis in the validation cohort suggesting that patients with haplotype combinations different from (CTC, ACG) have a somewhat similar prognosis. The (CTC, ACG) combination also remained an independent prognostic marker after the multivariate survival analysis in both cohorts.

The two haplotypes, CTC and ACG, might be linked to two independent genetic variations, both being of prognostic importance for different reasons and both acting in a dominant fashion. This would explain the inferior survival rates demonstrated for patients harboring both haplotypes compared to patients with only one of the haplotypes on one or both alleles. It should be pointed out that the associations to heterozygosity for −2578 C/A, −460 C/T and 405 G/C demonstrated in Table 2 and Table 3 are easily explained by the fact that the haplotype combination (CTC, ACG) will result in heterozygosity for these three SNPs. These genetic variations may lead to a differential influence on the tumor vasculature and thereby the risk of dissemination of tumor cells in the individual patients ultimately resulting in a difference in prognosis. 

The Korean study by Kim *et al.* [15] found the −2578 A, 405 G, 936 T haplotype to be associated with inferior survival rates. The Greek study by Dassoulas *et al*. [17] assessed the clinical importance of haplotypes based on the −2578 C/A, 405 G/C and 936 C/T SNPs but found no associations with survival or clinicopathological characteristics. Given the strong linkage between the −2578 C/A and the −460 C/T SNPs, the haplotype associated with inferior survival rates in the Korean study probably represent the ACG haplotypes from the present study. So despite rather large differences between our results on genotype level, some agreement seems to exist on the haplotype level.

Using genomic DNA derived from blood holds several technical as well as biological advantages compared to tumor DNA. Genomic DNA is easy to assess through a blood test compared to more invasive procedures such as biopsies. It is constant over time and not influenced by tumor biology or treatment. The quality of blood derived DNA is often higher than the tissue derived DNA thereby improving the quality of the analyses and the time spent on optimizing procedures.

The retrospective design of the present study has its limitations, but our conclusions are strengthened by the validation performed on an independent cohort. The study presented here with focus on CRC stages II and III is the largest one in the field so far but the sample size is still too small to draw any definite conclusions and prospective validation is still warranted to provide further evidence. 

## 3. Experimental Section

### 3.1. Study Population

This retrospective study on 486 patients, all Caucasians, consisted of a test cohort of 191 patients operated between January 1999 and December 2000 and a validation cohort of 295 patients operated between January 2002 and December 2008. Besides being operated at different time periods, the two cohorts also differed with regard to the patient materials available for analyses, formalin fixated paraffin embedded (FFPE) tissue in the test cohort and blood samples in the validation cohort. All patients underwent surgical resection of histologically verified adenocarcinomas of the colon or rectum at the Department of Surgery, Vejle Hospital, Denmark. The study only included patients with stage II and III disease. Patients having received preoperative chemoradiation of rectal cancer were not included. Furthermore, patients who died of post-operative complications or within one month from the operation were excluded (16 patients from the test cohort and 14 patients from the validation cohort). All patients from the two cohorts meeting these inclusion criteria were included in the study and hence no specific power calculations were applied to determine the sample size. Pre-treatment examinations included a chest X-ray and ultrasound or CT scan of the abdomen. Postoperatively the tumors were histologically classified and staged according to the pTNM system. Information regarding patient characteristics, relapse status and survival were based on patient records and registries. The study was approved by the Regional Scientific Ethical Committee for Southern Denmark according to Danish law, and informed consent was obtained from all patients enrolled in the study. 

### 3.2. Analysis of Single Nucleotide Polymorphism

Genomic DNA from the test cohort was derived from FFPE tissue. Fifty-μm sections of FFPE normal colorectal tissue were initially treated with xylene for deparaffinization and then washed two times in ethanol (99%). Samples were then incubated for two days in a lysis buffer and proteinase K at 56 °C. Genomic DNA from the validation cohort was derived from whole blood, which was obtained at the operation and stored at −20 °C. The DNA from both cohorts was isolated using the NucleoSpin® Tissue method according to the user manual (Machery-Nagel, Düren, Germany,) (http://www.mn-net.com/Portals/8/attachments/Redakteure_Bio/Protocols/Genomic%20DNA/UM_gDNATissue.pdf) and by the Maxwell® method (after 2006) according to the user manual (Promega Corporation, WI, USA) (http://www.promega.com/tbs/tm284/tm284.pdf).

The PCR analysis was performed using the ABI PRISM 7900 HT fast real-time PCR system (Applied Biosystem, Foster City, CA, U.S.A.). Commercial assays (which were functionally tested or validated from Applied Biosystem) were used for the analysis of the VEGF-A SNPs. Assay numbers and the approximate length of the amplification products (estimated by gel electrophoresis) were as follows: The VEGF-A −2578 C/A SNP; rs699947; C___8311602_10; 110/90 base pairs (depending on genotypes), the VEGF-A −460 C/T SNP; rs833061; C___1647381_10; 120 base pairs and the VEGF-A 405 G/C SNP; rs2010963; C___8311614_10; 100 base pairs.

In each well, of a 96 well microtiter plate, 10 μL were added consisting of 2 μL DNA and 8 μL of a mastermix containing the two primers and probes from the assay and universal PCR mix (also Applied Biosystems). Controls were analyzed along with the samples for final identification of the genotypes. The PCR analysis was conducted according to standard procedures with cycling conditions initially at 50 °C for 2 min and 95 °C for 10 min. This was followed by 40 cycles at 95 °C for 15 s (for denaturation), and 60 °C for 1 min (for annealing and elongation). Following end-point reading, genotypes were visualised on an allelic discrimination plot. Samples, from which the test results did not meet the quality value threshold, 95% for DNA derived from the blood samples and 98% for DNA derived from FFPE tissue, were diluted and reanalyzed. This was only necessary for a few samples. The SNP analysis was performed without knowledge of the clinical data.

### 3.3. Statistical Analysis

Fisher’s exact test was used for comparison between genotypes and patient characteristics. Chi-square statistics were used to test for Hardy-Weinberg equilibrium. The haplotypes and their frequencies were estimated using the PHASE program, version 2.1, which implements a Bayesian statistical method for reconstructing haplotypes from genotype data [24,25]. No patients had missing SNP data. Disease free survival was defined as the time from surgery until the first documented tumor recurrence or death. Overall survival was defined as the time from surgery until death. Survival curves were illustrated according to the Kaplan-Meier method and the logrank test was used to test for differences between the groups. Survival data from patients diagnosed with a new malignancy after their surgical resection for CRC (12 patients from the test cohort and nine from the validation cohort) were censored from the date of their new cancer diagnosis. This was done to prevent any possible bias related to the presence of a new cancer or the chemotherapeutic treatments used. Furthermore, survival data from seven patients were incomplete (five patients from the test cohort and two patients from the validation cohort) and were censored from the date of their last patient record. Cox regression method was used to analyze the independent prognostic importance of different markers. All statistical calculations were carried out using the NCSS statistical software (NCSS Statistical Software, Kaysville, UT 84037, USA, version 2007). P values <0.05 were considered significant, and all tests were two sided.

## 4. Conclusions

In conclusion, this validating study of nearly 500 patients from two independent cohorts with stage II and III CRC identified a genetic signature related to the prognosis of patients with stage II and III CRC based on genetic variations in the promoter and 5’UTR of the *VEGF-A* gene. Analysing haplotype combinations, we were able to identify a group with a rather favorable prognosis and a group in which adjuvant chemotherapy seems indicated. The possible benefit from such a treatment, however, cannot be assessed from the present results. This unfavorable haplotype combination remained an independent prognostic marker. Future studies should focus more on haplotype analyses because of higher degrees of consistency between studies. The haplotype combination approach calls for further investigation. 
